# Improving probe set selection for microbial community analysis by leveraging taxonomic information of training sequences

**DOI:** 10.1186/1471-2105-12-394

**Published:** 2011-10-10

**Authors:** Paul M Ruegger, Gianluca Della Vedova, Tao Jiang, James Borneman

**Affiliations:** 1 Department of Plant Pathology and Microbiology, University of California, Riverside, CA 92521, USA; 2 Department of Statistics, University of Milano-Bicocca, Milan, 20126, Italy; 3 Department of Computer Science and Engineering, University of California, Riverside, CA 92521, USA

## Abstract

**Background:**

Population levels of microbial phylotypes can be examined using a hybridization-based method that utilizes a small set of computationally-designed DNA probes targeted to a gene common to all. Our previous algorithm attempts to select a set of probes such that each training sequence manifests a unique theoretical hybridization pattern (a binary fingerprint) to a probe set. It does so without taking into account similarity between training gene sequences or their putative taxonomic classifications, however. We present an improved algorithm for probe set selection that utilizes the available taxonomic information of training gene sequences and attempts to choose probes such that the resultant binary fingerprints cluster into real taxonomic groups.

**Results:**

Gene sequences manifesting identical fingerprints with probes chosen by the new algorithm are more likely to be from the same taxonomic group than probes chosen by the previous algorithm. In cases where they are from different taxonomic groups, underlying DNA sequences of identical fingerprints are more similar to each other in probe sets made with the new versus the previous algorithm. Complete removal of large taxonomic groups from training data does not greatly decrease the ability of probe sets to distinguish those groups.

**Conclusions:**

Probe sets made from the new algorithm create fingerprints that more reliably cluster into biologically meaningful groups. The method can readily distinguish microbial phylotypes that were excluded from the training sequences, suggesting novel microbes can also be detected.

## Background

Microbes often exist in complex and dynamic communities that can have profound effects on the environments or hosts in which they live. Studies of microbial communities often begin with an assessment of which microbial taxa are present and in what numbers. These include studies that are primarily descriptive in nature or those seeking to make observations of broad trends or patterns in the taxonomic makeup of microbial communities in various niches [1-4].

Many methods currently exist to study microbial communities. These methods range from inexpensive, coarse-grained tools such as denaturing gradient gel electrophoresis (DGGE) [5] and terminal restriction fragment length polymorphism (T-RFLP) [6], to the significantly more expensive but more taxonomically accurate "gold-standard" of sequencing full-length 16S rRNA genes [7].

The coarse-grained methods are useful for examining changes in the predominant members of microbial communities from sample to sample, but the coverage is inadequate for some types of studies. For example, analysis of a community containing one million bacteria with T-RFLP might be depicted by a banding-pattern containing only 40 bands. Sequencing full-length 16S rRNA genes (~1550 bp) provides the highest available taxonomic resolution when an accurate "snapshot" of a microbial community is required. However, although costs are dropping, multi-sample longitudinal studies that employ full-length sequencing are still too expensive for many labs. High-throughput sequencing of portions of 16S rRNA genes currently provides the best compromise between accuracy and throughput, but due to the short read-lengths (~150-450 bp) these are limited to elucidating the population densities of a microbial community confidently only at the order taxonomic level and some confidence at the genus level, but very little confidence at the species level [3,4]. Moreover, because of this limitation, follow on studies where one endeavors to track population densities of specific bacterial species are often impossible.

This study focuses on improving an alternative method for analyzing population changes in microbial communities, termed oligonucleotide fingerprinting of ribosomal rRNA genes (OFRG) [8-10], which may be useful for studies requiring the analysis of many samples at higher taxonomic resolution than current high-throughput sequencing methods provide. To estimate the proportions of putative microbial phylotypes present in an environment, the OFRG method uses a set of 40 computer-designed 10-mer DNA probes, chosen from a set of training sequences, to hybridize against an array of sample-derived microbial rRNA gene clones [11]. The hybridization affinity of each probe/clone combination can be quantified and transformed into a 40-digit binary "fingerprint" for each clone. These experimentally-derived fingerprints can be clustered based on their similarity to the fingerprints of other clones in the array. Because similar fingerprints arise from similar rRNA genes and contain many thousands of clones, these clusters provide an estimate of the relative proportions of the various microbial taxa present in an environment.

Many computational methods exist to create microarray probe sets for conserved functional genes for microbial community analysis. These include such methods as Hierarchical Probe Design, PhylArray, HiSpOD, and CaSSiS [12-15]. These methods seek to design probes that are group- and/or sequence-specific. PhylArray also designs degenerate and non-degenerate probes to within-group polymorphisms in an effort to detect unknown bacteria in those groups. Once designed, probes can be affixed to a suitable microarray platform for later use.

These methods are unsuitable for our purposes because the OFRG method employs a fundamentally different strategy for discerning microbial assemblages than most microarrays. Rather than designing and affixing many hundreds or thousands of probes to an array, OFRG affixes the target genes to the array and sequentially hybridizes a small set of probes to it. Due to the nature of this paradigm, and the small size of probes (10-mers), it is neither necessary nor possible to find group-specific probes. Rather, the probes work together to distinguish taxonomic groups.

Choosing an optimal set of OFRG probes is challenging. We limit our laboratory experiments to 40 probes, as this provides a balance between technical constraints and the information each additional probe can provide. Therefore, the probes must be chosen carefully to maximize their utility. Previous work to create a probe set for OFRG built upon the work of Drmanac and Meier-Ewert [16-18] which investigated strategies to screen cDNA and BAC clone libraries with carefully chosen sets of probes. This concept was adapted to microbial community analysis by Borneman et al. [11] that used available 16S rRNA gene sequences as training data. A successful hybridization event of any probe to any gene is predicted during probe set design if the complete sequence of a probe is a substring of the gene's sequence. The formulation for probe set selection in [11] most pertinent to this work is termed the Maximum Distinguishing Probe Set (MDPS); to improve the ability of a probe set to distinguish bacterial phylotypes, we have modified the objective function employed by its simulated annealing algorithm to incorporate phylogenetic information.

As the name implies, the original MDPS attempts to create a probe set that produces a distinct binary fingerprint for all training sequences - maximizing the ability of the probe set to distinguish all sequences. Neither sequence similarity nor taxonomy is taken into account, however. Although the MDPS has been used successfully in several studies [8-10,19-22], the limitation of the MDPS from a biological perspective is that it considers all undistinguished clones (those having the same fingerprint) equally undesirable. By chance, fingerprints from similar DNA sequences do tend be similar or identical to each other, and fingerprints coming from dissimilar DNA sequences tend to be dissimilar to each other - but this is not always the case. More specifically, very divergent sequences having the same fingerprint are considered no worse than very similar sequences having the same fingerprint.

In the present study, we address this shortcoming of the MDPS with a new formulation for probe set selection termed the Maximum Fidelity Probe Set (MFPS) and a new processing pipeline for preparing the training data used by the MFPS.

## Methods

The new probe set selection method involves a change to the cost function within the simulated annealing algorithm used by Borneman et al. [11]. In addition, a processing pipeline was developed to prepare the training data. Within the simulated annealing algorithm, the MFPS is used to score each transient probe set using multiple penalty levels corresponding to the taxonomic levels of the training sequences. Recall that none of the probes are selected based on their specificity to or against any taxonomic groups. Rather, probe sets are evaluated as a unit. After many iterations of (random) probe substitution/probe set evaluation, a final probe set is output. Below we describe the new pipeline and cost function, highlighting the elements contributing to improved performance.

### Data Processing Pipeline

The processing pipeline prepares the training data for the cost functions to operate on. The three most important differences between the new and original processing pipelines are that in the new pipeline the sequences, *i*) have their hypervariable regions removed, *ii*) are clustered into species-like operational taxonomic units (OTUs) and, *iii*) are labeled with their OTU and higher-level taxonomic information.

**Figure 1:**
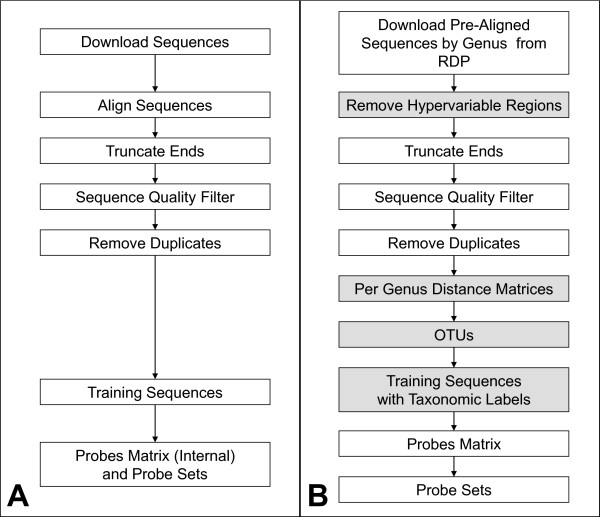
**Diagrams of the new and original processing pipelines**. Shown are the A) original processing pipeline and B) new processing pipeline for training sequences. The four main differences (shaded boxes) in the new are 1) sequences have their hypervariable regions removed, 2) distance matrices allow 3) grouping (< = 1% sequence difference) into Operational Taxonomic Units (OTUs), and 4) sequences are labelled with their taxonomic designations, as supplied by the Ribosomal Database Project (RDP).

Figures 1A and 1B show the new and original processing pipelines, respectively. The "original pipeline" was originally performed manually, step-by-step, with various software tools, as shown in Figure 1A. We automated it here in its essential aspects to facilitate comparisons to the new pipeline. The automated pipelines start with downloading pre-aligned rRNA gene sequences from the Ribosomal Database Project (RDP) on a per-genus basis. However, the new processing pipeline utilizes a "mask" sequence, supplied by RDP in each downloaded alignment file, that denotes the location of hypervariable regions within the alignment (see first shaded box, Figure 1B); these are used in combination to remove the hypervariable regions in the sequences, as any probes designed to bind in those regions would hybridize to only a few taxonomic groups and thus provide little to no help in distinguishing most other taxonomic groups.

The pre-aligned sequences also simplify the creation of distance matrices used to create OTUs, and the task of truncating the ends of the sequences. It is useful to truncate the ends to create more consistent training data, as their lengths can vary due to the presence of partial gene sequences stored in the RDP database. To do so, we truncated ten nucleotide positions "inward" of the locations of two highly conserved primer regions (27 F - AGAGTTTGATCMTGGCTCAG and 1392R - ACGGGCGGTGTGTRC) that we use in the lab, thus leaving only the portions of the 16S rRNA gene intended as the target for probes. For both pipelines, a sequence was considered too short and rejected if there was an end gap in the alignment after truncation and the truncated section from that end contained only gaps. No attempt was made to discover or correct for sequence errors in canonical bases. However, sequences with ambiguous bases, and duplicate sequences, were removed.

Per genus distance matrices are created from the aligned sequences. Per genus OTUs are then created from the distance matrices using the program MOTHUR [23] (second and third shaded boxes, Figure 1B). All OTUs were made with a minimum sequence similarity of 99%. The OTU, genus and phylum information was then concatenated to the corresponding DNA sequences.

Both processing pipelines then create a probe matrix from the training sequences. The matrices are comprised of a list of candidate probes (rows) and their putative binding ability to each of the training sequences (columns), and include the taxonomic information of each sequence (last shaded box, Figure 1B). Making a matrix once and saving it allows the cost functions to operate more efficiently. Constructing the probe matrix begins by creating a list of all 10-mers that occur at least once in the training sequences. This list can grow to over 750, 000 probes, depending on the size of the data set, and must be reduced due to practical considerations of computational time and memory limitations. The size reduction was accomplished by a filtering step to keep only 1000 of the most highly conserved probes (based on how many OTUs a probe is found in). For each probe/sequence combination in the probes matrix, a 1 or 0 denotes whether the probe sequence was found in or not found in the training sequence, respectively. Taxonomic data are converted to numbers and added to the probes matrix so it is accessible to the MFPS. Our implementation of the original MDPS uses the same matrix for probe and binding information but the taxonomic information is ignored.

To compare the two pipelines, we made training sequences and probe matrices with both. The training data from the original pipeline differs from the new in that the hypervariable regions were not removed from the sequences prior to making the probe matrix, and the list of candidate probes in the two matrices are not identical because of this. To examine just the pipeline's effect on probe sets, apart from any added benefit of using taxonomic information, we employed only the original MDPS algorithm, making probe sets of sizes 20, 30, 40, 60 and 80 probes per probe set.

Note that this comparison of the two pipelines is the only experiment where probe sets were made from the automated original pipeline. All other experiments used probe sets made from the new pipeline.

### Maximum Fidelity Probe Set (MFPS)

By employing a heuristic strategy, the MFPS scores each transient probe set using multi-level penalties corresponding to the taxonomic levels of the training sequences. By doing so, it addresses the main weakness of the cost function in the MDPS, which attempts to choose a probe set that creates a distinct binary fingerprint for each training sequence without regard to sequence similarity or taxonomy.

To adequately explain the MFPS, we first define several terms. A *simulated fingerprint *is a binary vector of *k *digits representing the putative hybridization pattern of *k *DNA probes on a DNA sequence of interest. For our purposes, the sequences we are interested in are bacterial 16S rRNA genes and the DNA probes are 10 bases long. If the sequence of a probe occurs exactly in the sequence of a gene, we assume it would hybridize to the gene in a real hybridization experiment, and if it does not occur exactly we assume it would not hybridize. Therefore, we place a 1 or 0 into each of the *k *characters of the simulated fingerprint of a gene sequence to denote a putatively successful or unsuccessful hybridization event for each of the *k *probes of a probe set.

A *distinct fingerprint *is simply a single representative of a group of identical simulated fingerprints produced by a probe set P in a set of sequences S. It is useful in determining a probe set's quality score - its *fidelity*.

The *fidelity *of a probe set is determined from the fidelity of the distinct fingerprints it produces. It is used to gauge the quality of a probe set and is explained as follows. If a distinct fingerprint *f *is produced by probe set P on one or more sequences in taxonomic group *γ *in a set of sequences S, and *f *is *not *produced in any other taxonomic group at the same level as *γ*, then *f *is said to have high fidelity - a desirable trait. Conversely, if fingerprint *f *is produced on one or more sequences outside of taxonomic group *γ *in S, then *f *is said to have low fidelity. Additionally, the more groups outside of *γ *where fingerprint *f *is produced, the lower its fidelity is said to be.

Note that fidelity is always associated with a taxonomic level. For instance, a distinct fingerprint *f *may have low fidelity at the OTU level (if it occurs in the sequences of two or more OTUs) yet have high fidelity at the genus level (if it occurs in the sequences of only one genus). The aim of the MFPS is to select a set of probes that together produce high-fidelity distinct fingerprints at the taxonomic level(s) desired. If this can be achieved, distinct fingerprints arise within biologically meaningful taxonomic groupings and can be used as proxies for them. To that end, probe sets are evaluated in the MFPS by the cost function,

$$c=\frac{1}{2}\overset{N}{\underset{f=1}{\sum }}\overset{3}{\underset{i=1}{\sum }}P_{i}\gamma_{i,f}\left(\gamma_{i,f}-1\right)$$

where *C *is the total cost, N is the number of distinct fingerprints produced by the probe set on the training sequences, *i *is one of three taxonomic levels (we used OTU, genus and phyla but others could be used), *f *is an individual distinct fingerprint, *γ_i, f _*is the number of taxonomic groups where *f *occurs at taxonomic level *i*, and *P_i _*is the penalty (for low-fidelity fingerprints) at taxonomic level *i*. Note that if a distinct fingerprint is found in only one taxonomic group (*γ_i, f _*= 1) then no penalty will accrue to the probe set from that fingerprint. This cost function of our MFPS replaces the cost function in the simulated annealing algorithm used by Borneman et al. [11].

Note that the cost function allows one to vary the penalty level for up to three taxonomic levels simultaneously. Experiments to find optimal penalty settings were conducted by systematically varying them and comparing the results. These experiments were conducted with probe sets containing 20, 30, 40, 60 and 80 probes. For each experiment, at each penalty level and probe set size, one hundred probe sets were created using the MFPS and MDPS cost functions.

When cross-validation was performed, we used a variation of 5-fold cross-validation. Instead of the traditional 80% training/20% validation, we chose to use a 20% training/100% validation strategy. Due to the nature of one of our evaluation metrics, this strategy allowed us to better compare the results of other tests where we used 100% of the training data to make and evaluate probe sets. The 20%/100% also provides a more stringent test of probe set design than 80%/20%. All cross-validation data shown are an average of 5-fold results.

### Evaluation Metrics

Two evaluation metrics are used to compare the two pipelines and cost functions. The first metric is termed the High Fidelity Ratio (HFR), which is the ratio of distinct high-fidelity fingerprints produced by probe set P (on validation data) and the total number of distinct fingerprints produced by P on the same data. In essence, the HFR is a measure of how closely the simulated fingerprints arising from a probe set on the sequences are representing real OTUs and genera. Importantly, the HFR metric is comparable across probe sets; because the raw scores of the cost functions are dependent upon the penalty levels chosen, as well as the number of probes in a probe set, they cannot be used to compare probe sets made with different penalty levels or different numbers of probes. Note that a probe set can have one HFR for each taxonomic level evaluated. In our experiments, we examine OTU and genus HFRs only, as phyla HFR automatically improves when lower-level fidelity improves.

The second evaluation metric we used was the average pairwise sequence distance of each low-fidelity distinct fingerprint in a probe set. Rather than a single number, this metric is shown as a line graph and was constructed as follows. For each low-fidelity distinct fingerprint *f *in probe set P, we take all sequences having *f *and compute their average pairwise sequence distance. Bin each average into bin sizes of 1% difference. Continue this for as many probe sets as were made for the experiment (usually 100) and graph the overall averages for each bin. Note that it is not necessary to examine the high-fidelity distinct fingerprints in this way as they cannot, by definition, exceed the OTU cutoff threshold of 1% sequence difference.

Both new and original processing pipeline scripts were written in Perl. The probe set selection software was written in C. All software is open source and is available for download at https://github.com/ofrg/OFRG-Probe-Set-Design. Sequences and taxonomic information were downloaded from the Ribosomal Database Project (Release 10, Update 14) [24].

### Effect of Sequencing Read Length on Taxonomic Resolution

We performed an analysis to explore the effect of sequencing read lengths of 16S rRNA genes that would be necessary to discriminate sequences at the genus level using the latest RDP Classifier (RDPC) [25] version 2.3, downloaded from SourceForge. Simulated reads, of lengths 200 bp up to 1400 bp (in 200 bp increments), were extracted from (already classified) full-length RDP 16S rRNA gene sequences, beginning from several universal bacterial primer sites. Sequences used met the same quality requirements of our data processing pipeline described above (i.e., they must be of sufficient length and not contain ambiguous bases). For each read length and primer start point, 40, 000 reads were selected randomly and processed through the RDPC, which classifies the reads and calculates a confidence score for each taxonomic level it assigns. To assess a simulated read's classification accuracy, we considered it correctly classified if its classification matched the classification of the full-length sequence from which it came, regardless of the confidence level calculated by RDPC.

### A Practical Consideration for Wet Lab Hybridizations

In the event the hybridization behavior of one or more probes is deemed to be unsatisfactory in laboratory conditions, they can be replaced; the program is capable of retaining or avoiding specific probes when making a probe set. In our experiments, only one of 40 probes performed poorly due to high background values.

## Results and Discussion

### Comparison of Data Processing Pipelines

We compared the new and original processing pipelines using the High Fidelity Ratio (HFR) metric and the Maximum Distinguishing Probe Set (MDPS) of Borneman et al. [11]; the MDPS does not use taxonomic information so any differences in the results can be attributed solely to the pipelines.

**Figure 2:**
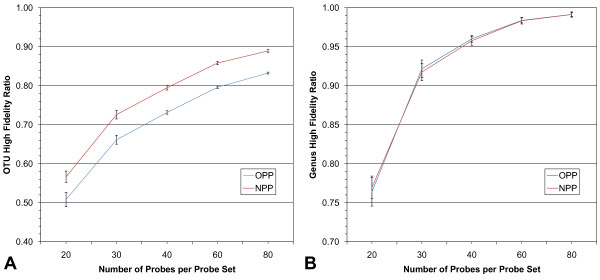
**Processing pipeline effect on fidelity**. The effect on fidelity of the new (NPP) and original processing pipelines (OPP) in a range of probe set sizes using only the MDPS with the 20%/100% training/validation data. A) OTU fidelity appears higher in the new. B) Genus fidelity shows little difference between the two pipelines. Error bars are the standard deviations of 100 probe sets per data point. Note: for display purposes, the y-axis scales are not identical in A and B.

The new processing pipeline shows an improved OTU HFR over the original pipeline in probe sets ranging in size from 20 - 80 probes (Figure 2A). The improvement is approximately the same across the range of probe set sizes. The poorer performance of the original pipeline is most likely due to the increased number of OTUs created by it, as having more OTUs will tend to lower the odds of successfully distinguishing them. There were 203, 218 sequences distributed in 34, 701 OTUs using the new pipeline and 216, 414 sequences distributed in 52, 983 OTUs with the original. The difference in the number of sequences in the pipelines arises when removing duplicates; hypervariable regions are not removed in the original pipeline, which increases the odds a that sequence will be unique by at least one base. The average OTU sizes for the new and original pipelines are 5.86 and 4.08 sequences, respectively. The increased numbers of OTUs, in turn, is due to both the greater number of sequences allowed into the training set by the original pipeline and the presence of the hypervariable regions, which often makes the average pairwise sequence distances greater and thus leads to more and smaller OTUs. The genus-level HFRs were very similar to each other, however, with a slightly better score seen in the original pipeline with probe sets of size 30 and 40 (Figure 2B). The high overall similarity of HFR scores at the genus level is reflective of the fact that the number of genera represented in the data from both pipelines is the same; genus designations are made by the RDP database, unlike OTU designations that are made by the processing pipelines. The slightly better genus-level HFR in the original pipeline is thus either due to the presence of hypervariable regions or the increased numbers of training sequences per genus.

Regarding the hypervariable regions, the rationale for removing them in the new pipeline is that candidate probes arising from these areas may target only a narrow range of taxa and may thus be less informative than more conserved probes - yet they may be common enough in the training data (where some taxa may be overrepresented) to be chosen for a final probe set. By removing the hypervariable regions, the average pairwise sequence similarities will tend to increase - a situation that can lead to the creation of larger and fewer OTUs for any given similarity threshold. Therefore, we set the inclusion threshold for OTUs to 99% sequence similarity, which serves as a relatively conservative target and benchmark for creating and evaluating probe sets.

The new pipeline's contribution to better probe sets is supportive and indirect. It enriches the pool of more informative candidate probes and attaches the taxonomic information of the sequences for the MFPS cost function to operate on. In addition, the new pipeline facilitates updating an OFRG probe set with the latest sequence information. With relatively minor modifications, the pipeline could be adapted for use on ribosomal (or other) genes of different microorganisms.

### Optimizing Penalty Levels of the MFPS Cost Function

Our primary goal was to create a probe set with the highest possible OTU fidelity, as this maximizes the number of fingerprints that represent real OTUs. A secondary goal was to minimize low fidelity fingerprints at the phylum level, as these represent the worst cases. A tertiary goal was to improve the behavior of low fidelity fingerprints by minimizing the average pairwise sequence distance metric.

The new cost function of the MFPS is capable of employing up to three penalty settings corresponding to three levels of taxonomic information supplied in the training data (we used OTU, genus and phylum). As mentioned previously, we found that using a phylum penalty was unnecessary to achieve our secondary goal of improving phylum HFR, so it was always set to zero when making probe sets for the MFPS; phylum HFR rose to nearly 100% when OTU fidelity was optimized.

**Figure 3:**
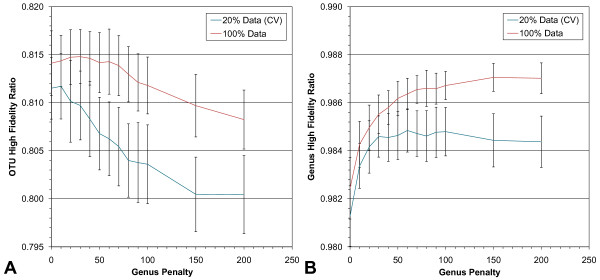
**Optimizing the genus penalty for the MFPS cost function**. The genus penalty was varied from 0 to 200 while holding the OTU penalty at 1. A) OTU fidelity rises slightly as the genus penalty increases from zero and then declines. B) Genus fidelity rises sharply and then plateaus. A penalty of 10 achieves the highest OTU fidelity in the 20% cross-validation data (CV) and OTU fidelity is highest in the 100% data set at a penalty of 30. Error bars are the standard deviations of 100 probe sets per data point. Note: for display purposes, the y-axis scales are not identical in A and B.

With the OTU penalty set to 1, Figure 3 shows how the HFR metric is affected as the genus penalty increases relative to the OTU penalty. In each panel (A and B) two results are shown. The blue lines show the average HFR scores of 100, 5× cross-validation probe sets per point, and the red lines show the average scores of 100 probe sets per point but using 100% of the data for training and validation.

Notice in Figure 3A that there is a slight increase in the OTU HFR before beginning a downward trend. This effect is seen in both 100% and 20% cross-validation (CV) probe sets, with the 20% cross-validation reaching a maximum at a genus penalty of 10 and the 100% sets reaching a maximum at a genus penalty of 30. Figure 3B shows how the genus HFR is affected as the genus penalty increases. This number rises and eventually plateaus, with more variation and a lower plateau seen in the 20% cross-validation data.

An OTU penalty of 1 and a genus penalty of 30 for the MFPS were chosen as optimal for a comparison to the MDPS. Our rationale for choosing a genus penalty of 30 was as follows. The initial rise in OTU fidelity makes intuitive sense because the increasing genus penalty improves the chances a distinct fingerprint will occur in only one genus - but if more distinct fingerprints are occurring in only one genus it becomes more likely some will also occur in only one OTU within that genus. However, as the genus penalty increases further and the total penalty score for a candidate probe set becomes dominated by any mistakes in genera classification, the MFPS begins to sacrifice OTU fidelity for better genus fidelity. Finally, the peak OTU fidelity occurs at a lower genus penalty level in the smaller 20% cross-validation data than in the 100% data set (10 and 30, respectively), suggesting that the size and/or makeup of the training data influences the optimal genus penalty level.

This led us to conclude that the larger the data set the farther to the right the OTU maximum might appear. And, since we planned to order a set of probes for laboratory use on environmental samples, we should design them with a large data set in mind. Nevertheless, choosing a genus penalty above 30 would be an extrapolation.

The risk of overfitting may be higher when using the full data set, but since it is impossible to predict what bacteria a sample will contain, it is not clear how we can know we have or have not over-fit the data. Also, based on the severe tests of removing whole phyla (see Effect of Removing Whole Phyla section below) and using only 20% cross-validation data evaluated on 100%, the solution-space appears to be broad, and good solutions abundant, even if an optimal one is elusive.

### Comparison of MFPS and MDPS Cost Functions

**Figure 4:**
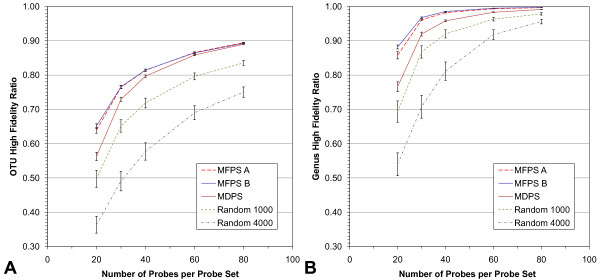
**Comparison of the MFPS and MDPS using the High Fidelity Ratio (HFR)**. HFRs at the A) OTU level and B) genus level are shown. Both MFPS A and MFPS B (OTU penalties set to 1, and genus penalties set to 0 and 30, respectively) score higher than the MDPS in all probe set sizes examined but the difference narrows from n = 20 to n = 80 probes. Randomly chosen probe sets perform less well. The Random 1000 probe sets selected probes from the top 1000 most conserved probes and the Random 4000 from the top 4000. The MFPS and MDPS also selected probes from the 1000 most conserved probes. Error bars are the standard deviations of 100 probe sets per data point.

Figure 4 shows the performance of the MFPS and MDPS cost functions, using the HFR metric, with probe sets containing between 20 and 80 probes. We include two versions of MFPS penalty settings to highlight the source of improvements over the MDPS. MFPS A and B (genus penalties of 0 and 30, respectively), show very similar OTU HFR scores for all probe set sizes, while MFPS B edges out MFPS A in genus HFR. MFPS A scores higher than the MDPS yet similarly to MFPS B in all probe set sizes examined, suggesting that most of the benefit in fidelity stems from the OTU penalty via the OTU clustering strategy employed by the MFPS. The difference between MFPS and MDPS is most pronounced in probe sets of size 20 and gradually narrows up to probe sets of size of 80. For OTU HFRs, the scores at n = 80 are nearly identical, but for genus HFRs the MFPS still shows a slightly improved performance over the MDPS.

As a control, probe sets were created randomly from one of two differently-sized probe matrices - either 1000 probes (the same one used to compare the cost functions) or 4000 probes, and are also included in Figure 4. The HFRs of the MFPS and MDPS are indeed higher than both random probe sets. Interestingly, the HFRs of random probe sets from the 4000 probe matrix were much lower than the probe sets made from the 1000 probe matrix.

To explain this difference, recall that the random 1000 probe sets contain probes from the top 1000 most conserved probes and the random 4000 from the top 4000. The higher HFR scores observed from the smaller probe matrix therefore suggests these are somehow more informative taxonomically.

Our laboratory experiments will be done with a set of 40 probes, as this is a practical maximum and provides very high (theoretical) fidelity. Using 40 probes, genus-level HFR is over 98% and OTU-level HFR is over 81%. It is also worth noting that with 40 probes the majority (~55%) of low-fidelity distinct fingerprints (which comprise less than 19% of all distinct fingerprints) occur in only two OTUs, but within the same genus.

### Average Pairwise Sequence Distances

**Figure 5:**
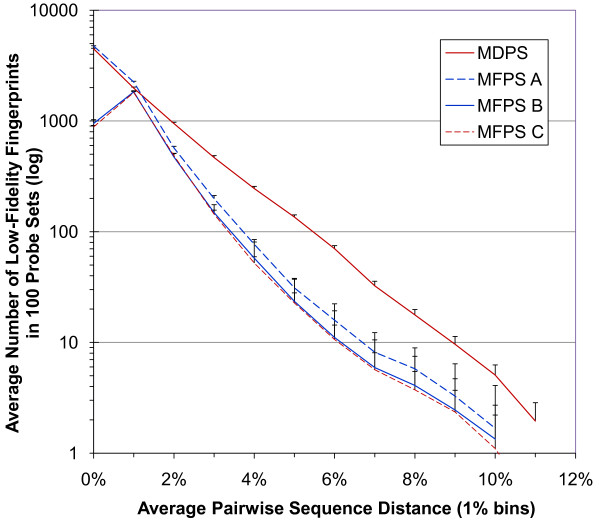
**Average pairwise sequence distance metric**. This metric focuses on how inaccurate a probe set's low-fidelity fingerprints are. Lower scores are better. The graph was constructed as follows. For each low-fidelity distinct fingerprint of a probe set, the average pairwise sequence difference between its underlying DNA sequences was determined. A count of how many fingerprints within each binned (1% increments) average was kept. Each point represents the average count of each bin for 100 probe sets. MFPS A (OTU and genus penalties set to 1 and 0, respectively) is superior to MDPS except for having a few more fingerprints from 0% to 1%; scores in this range are from highly similar sequences but from OTUs in different genera. MFPS B (OTU and genus penalties set to 1 and 30, respectively) shows further improvement in distances greater than 1%, but unlike MFPS A or MDPS, has markedly fewer low-fidelity distinct fingerprints with sequence distances from 0% to 1%. The improvement in distances greater than 1% is the same windfall seen in HFR scores when the genus-level penalty was set to 30 (see Figure 3). MFPS C (OTU and genus penalties set to 1 and 200, respectively) shows only a small improvement over MFPS B. Error bars (showing upper bars only for better visibility) are standard deviations from 100 probe sets.

The average pairwise sequence distances results are shown in Figure 5. Unlike the High Fidelity Ratio, which is a measure of the taxonomic accuracy of a probe set, this metric focuses on the inaccuracy of a probe set's low-fidelity fingerprints, measuring the dissimilarity of the underlying DNA sequences from which they arose. Figure 5 reveals a considerable overall improvement of the MFPS over the MDPS, as well as the effects different penalty settings have in the MFPS. To evaluate the two cost functions with this metric, we compared their results using three different penalty schemes for the MFPS.

Compared to the MDPS line, MFPS A (OTU and genus penalties set to 1 and 0, respectively) is superior except for having a few more sequences from 0% to 1%. The improved scores beyond 1% difference reflect the tendency of all distinct fingerprints (high and low fidelity) to more closely pattern real taxonomic groups; even if they do occur in more than one OTU, they tend to occur in more similar sequences. Likely for the same reason, the MFPS A performs more poorly from 0% to 1%. These scores are from highly similar sequences in different OTUs but presumably from different genera (otherwise they would have been grouped into the same OTU). This phenomenon is consistent with the fact that there was no genus-level penalty imposed in MFPS A.

MFPS B (OTU and genus penalty levels set to 1 and 30, respectively) shows further improvement in distances greater than 1%, but unlike MFPS A or MDPS, has markedly fewer low-fidelity distinct fingerprints with distances less than 1%. The latter is clearly an effect stemming from the genus-level penalty imposed during probe set creation; now, probe sets are shepherded away from these "near-misses." The improvement in distances greater than 1% is the same windfall seen in HFR scores when the genus-level penalty was set to 30 (see Figure 3A).

MFPS C (OTU and genus penalty levels set to 1 and 200, respectively) shows only a small improvement over MFPS B, and comes at the expense of OTU fidelity (see Figure 3A). Such a small improvement, along with the plateauing of genus fidelity above a penalty of 150 (see Figure 3B), suggests we are at or near the limit of n = 40 probe sets produced by the MFPS.

### Effect of Removing Whole Phyla

To examine how the fidelity of probe sets might behave if sequences from unknown phyla are encountered, MFPS and MDPS probe sets were made after sequentially removing several of the largest phyla, each ranging in size from approximately 10% to 33% of all training sequences.

**Figure 6:**
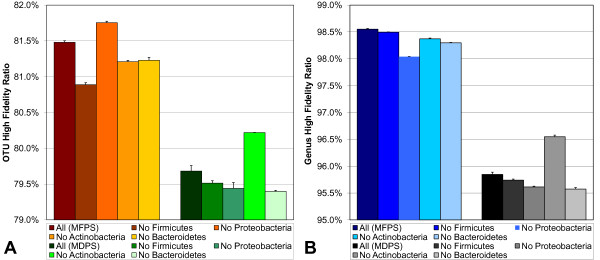
**Fidelity effect when removing whole phyla from training data**. A) OTU fidelity. B) Genus fidelity. Although both MFPS and MDPS are negatively affected generally by removing whole phyla, the effect is relatively minor, and the MFPS outperforms the MDPS. OTU HFR actually goes up in the MFPS and MDPS when the phyla Proteobacteria and Actinobacteria are removed, respectively. Genus HFR also goes up in the MDPS when Actinobacteria are removed. However, genus HFR decreases in the MFPS when Proteobacteria are removed. The proportions of all training sequences each tested phylum contributes are: Firmicutes (33.5%), Proteobacteria (25.7%), Actinobacteria (24.1%) and Bacteroidetes (9.6%). Error bars are the standard deviations of 100 probe sets.

Evaluations of the probe sets were performed with all phyla included. The results shown in Figure 6 indicate that although both MFPS and MDPS are negatively affected generally, the effect is relatively minor, and the MFPS outperforms the MDPS.

Interestingly, OTU HFRs went up in the MFPS and MDPS when the phyla Proteobacteria and Actinobacteria were removed, respectively. When looking at the genus HFRs for these phyla, removing Proteobacteria does not improve in MFPS, yet HFR still improves in the MDPS when removing Actinobacteria. It is not clear why an increase of HFR scores would occur when removing a phylum before making probe sets, other than that something in these phyla are causing the algorithms to become confused, perhaps trapping them in a local minimum.

### Positional Bias of Probes in MFPS and MDPS

**Figure 7:**
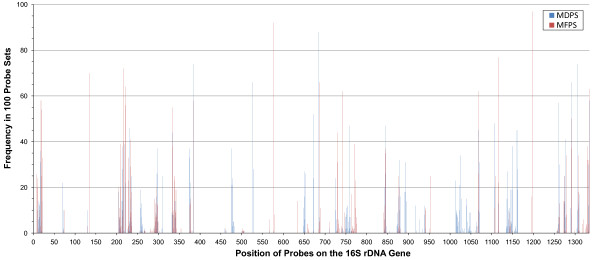
**Positional bias of probes from MFPS and MDPS**. This graph was constructed by finding the putative binding positions (on the 16S rRNA gene) of all probes in 100 probe sets of size n = 40 probes and plotting the frequency where they occurred at each position for both cost functions. Although probes arising from some positions appear to be chosen by both cost functions, there are several positions that appear to be favored by the MFPS or MDPS, sometimes exclusively.

We were curious if the probes chosen by the two cost functions would show any positional bias on the 16S rRNA gene sequence. Figure 7 was constructed by finding the starting positions of all probes in 100 probe sets of size 40 and plotting the frequency they occurred at each position for both cost functions. Although probes arising from some positions appear to be chosen by both cost functions there are several positions that appear to be favored by the MFPS or MDPS, sometimes exclusively.

The regions favored by the MFPS suggest these may tend to be more conserved within taxonomic groups, whereas the regions favored by the MDPS may tend to be less conserved within the same groups. Alternatively, because probes in a probe set are chosen to work together to provide information about the sequences, there may be some kind of complex within-group conservation between the regions being favored. More investigation would need to be performed to determine if there was some underlying biological significance to these patterns.

### Effect of Sequencing Read Length on Taxonomic Classification

**Figure 8:**
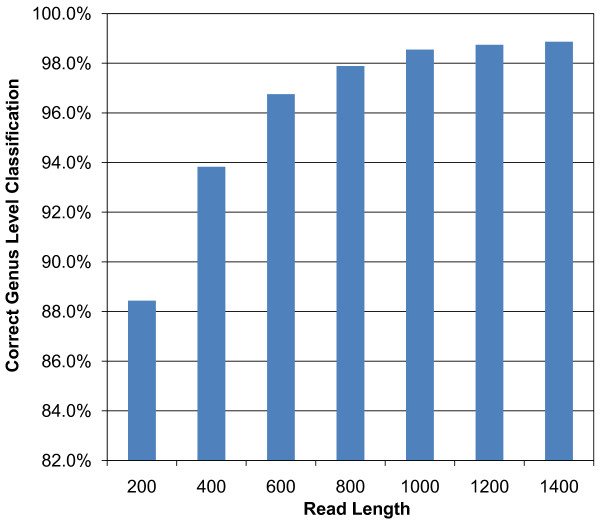
**Effect of Sequencing Read Length on Taxonomic Classification**. Simulated reads of various lengths (200 bp - 1400 bp) were taken from full-length 16S rRNA gene sequences (starting from several universal bacterial primer sites) and classified with the RDP Classifier. Read classifications were considered correct if they matched the taxonomic group of their full-length counterparts. Values are averages. See Additional File 1 for detailed classification percentages at each primer site and read length shown in this figure.

To provide some information comparing the effect of sequences of different read lengths and their correct classification at the genus level, we performed a simulated sequencing study. Starting with full-length 16S rRNA gene sequences classified by the RDP Classifier, we extracted simulated reads of various lengths (200 bp - 1400 bp, in 200 bp increments) and classified them with the RDP Classifier. Reads were considered correctly classified if they were classified into the same taxonomic group as their full-length counterparts. Figure 8 (a summary of Additional File 1) shows the average correct classification percentages of the various read lengths used. Read lengths above 800 bp are classified accurately about 98% or more of the time, while accuracy drops to a low of about 88% for 200 bp reads.

Although the results of this analysis indicate that read lengths of ~800 bp would be necessary to obtain a result similar to that achieved by the probe sets designed by our new algorithm (Figure 4B), we emphasise that OFRG and nucleotide sequencing are very different technologies and comparisons between them must be made carefully. OFRG's strengths will be advantageous for only certain types of studies, for example, when investigators endeavor to identify specific bacteria that correlate with a functional parameter such as disease. In this application, OFRG is used to obtain the population densities of unidentified OTUs. If any OTUs correlate with disease, they are deemed worthy of further study, and OFRG provides a way to extract and sequence their near full-length 16S rRNA genes. Obtaining these relatively long sequences allows for better phylogenetic identification and makes follow on studies such as sequence-selective quantitative PCR more feasible [21,22].

### Algorithm Performance

The 1000 probe matrix we used for most experiments is 391 MB in size. The RAM used by the probe set design program, which requires loading the matrix into memory when creating probe sets, was 410 MB. For our experiments, we set a parameter that causes the program to output only the single best probe set out of ten. Each 40 probe set produced this way takes ~2 h 40 m on a single 2.5 GHz Intel^® ^Xeon^® ^E5420 CPU.

### Future Directions

One future improvement in the MFPS would be to take into account more complex interactions between the probe and DNA strands. It is known, for instance, that in real hybridization experiments a probe can hybridize with varying degrees of affinity depending on several factors. These factors include being able to hybridize at a detectable level even when there is a single nucleotide mismatch between the probe and DNA, or less strongly than expected with a perfect match because of sequence-dependent steric effects.

Incorporating real probe hybridization behavior into an objective function would almost certainly increase the fidelity of probe sets produced by it. Unfortunately, small probe hybridization behavior is not well characterized and it is not currently possible to accurately predict binding affinity for all possible variations, which may negatively affect the specificity of the method. Thus, this remains a weakness of the current method.

However, although precise prediction of hybridization affinity is currently impossible, we have observed that the 10-mer probes used in our experiments do generally follow our simple model of hybridization behavior. That is, the case of a perfect match between a probe and DNA strand usually produces a brighter signal (indicating higher binding affinity) than cases where one or more mismatches are present. Importantly, though mismatch cases can result in intermediate binding affinity, experiments indicate these are often distinguishable from their perfect match counterparts, and even other types of mismatches. Accordingly, we have developed strategies that classify these data [26]. In addition, prior utilization of OFRG-based analyses have identified numerous differences in phylotype population densities that have been verified by sequence-selective qPCR analysis [21,22].

## Conclusions

With its multi-level penalty scheme the MFPS improves the quality of OFRG probe sets as measured by two biologically relevant metrics: fidelity and sequence distances. By pre-clustering training sequences into biologically meaningful groups, and then choosing probe sets based on how closely their resultant fingerprints represent those groups, we improve the odds that they will. We also show that the underlying sequences of low fidelity fingerprints are more similar to each other than in the original MDPS.

The MFPS has potential advantages over current high-throughput sequencing technologies in discriminating microbes at or near the species level. Attempts have been made to enumerate microbial phylotypes with the relatively small sequencing reads from the 454 and Illumina platforms (~450 bp and ~150 bp, respectively) by taxonomically classifying them, but are so far only able to do so confidently at the order level, and some confidence at the genus level [3,4]. This is because the taxonomic information in the 16S rRNA gene is not wholly contained in any contiguous portion of the gene targeted by these technologies, and accurate assembly of small reads from mixed bacterial communities into larger, single-species contigs is impossible due to the gene's conserved nature across species. In contrast, OFRG probes chosen by the MFPS are not restricted to a contiguous portion of the gene, but act in concert to target taxonomically important regions, providing near species-level (OTU) resolution in most cases, and genus-level resolution in nearly all cases (81% and 98%, respectively).

The taxonomic resolution of the method is robust; completely removing large taxonomic groups from training sequences had only a small negative effect on the ability of probe sets to distinguish those groups. These results, and the 20% cross-validation (CV) results, strongly suggest novel microbes can be detected by the method.

## Authors' contributions

PMR conceived of the new cost function and designed the study, developed the pipeline software, performed analysis and wrote the paper. TJ and JB contributed to the study design. GDV developed the probe set selection software. GDV, TJ and JB contributed to analysis and manuscript writing. All authors read and approved the final manuscript.

## Supplementary Material

Additional file 1 **Effect of Sequencing Read Length on Taxonomic Classification Detail**. This file contains the detailed results of the simulated read length on taxonomic classification study shown in Figure 8. Simulated reads, of lengths 200 bp up to 1400 bp (in 200 bp increments), were extracted from (already classified) full-length RDP 16S rRNA gene sequences, beginning from several universal bacterial primer sites. Sequences used met the same quality requirements of our data processing pipeline (i.e., they must be of sufficient length and not contain ambiguous bases). For each read length and primer start point, 40, 000 reads were selected randomly and processed through the RDP Classifier (RDPC) version 2.3. We considered a read correctly classified if its classification matched the classification of the full-length sequence from which it came, regardless of the confidence level calculated by the RDPC. Click here for file

## References

[B1] Liu Z Lozupone C Hamady M Bushman FD Knight R Short pyrosequencing reads suffice for accurate microbial community analysis Nucleic Acids Research 2007 35 e120 e120 10.1093/nar/gkm541 17881377 PMC2094085

[B2] Caporaso JG Lauber CL Walters WA Berg-Lyons D Lozupone CA Turnbaugh PJ Fierer N Knight R Microbes and Health Sackler Colloquium: Global patterns of 16S rRNA diversity at a depth of millions of sequences per sample Proc Natl Acad Sci USA 2010 10.1073/pnas.1000080107 PMC3063599 20534432

[B3] Wu GD Lewis JD Hoffmann C Chen Y-Y Knight R Bittinger K Hwang J Chen J Berkowsky R Nessel L Li H Bushman FD Sampling and pyrosequencing methods for characterizing bacterial communities in the human gut using 16S sequence tags BMC Microbiol 2010 10 206 10.1186/1471-2180-10-206 20673359 PMC2921404

[B4] Bartram AK Lynch MDJ Stearns JC Moreno-Hagelsieb G Neufeld JD Generation of Multimillion-Sequence 16S rRNA Gene Libraries from Complex Microbial Communities by Assembling Paired-End Illumina Reads Applied and Environmental Microbiology 2011 77 3846 3852 10.1128/AEM.02772-10 21460107 PMC3127616

[B5] Muyzer G DGGE/TGGE a method for identifying genes from natural ecosystems Current Opinion in Microbiology 1999 2 317 322 10.1016/S1369-5274(99)80055-1 10383868

[B6] Schütte UME Abdo Z Bent SJ Shyu C Williams CJ Pierson JD Forney LJ Advances in the use of terminal restriction fragment length polymorphism (T-RFLP) analysis of 16S rRNA genes to characterize microbial communities Appl Microbiol Biotechnol 2008 80 365 380 10.1007/s00253-008-1565-4 18648804

[B7] Frank DN St Amand AL Feldman RA Boedeker EC Harpaz N Pace NR Molecular-phylogenetic characterization of microbial community imbalances in human inflammatory bowel diseases Proc Natl Acad Sci USA 2007 104 13780 13785 10.1073/pnas.0706625104 17699621 PMC1959459

[B8] Valinsky L Della Vedova G Scupham AJ Alvey S Figueroa A Yin B Hartin RJ Chrobak M Crowley DE Jiang T Borneman J Analysis of bacterial community composition by oligonucleotide fingerprinting of rRNA genes Appl Environ Microbiol 2002 68 3243 50 10.1128/AEM.68.7.3243-3250.2002 12089000 PMC126790

[B9] Valinsky L Della Vedova G Jiang T Borneman J Oligonucleotide fingerprinting of rRNA genes for analysis of fungal community composition Appl Environ Microbiol 2002 68 5999 6004 10.1128/AEM.68.12.5999-6004.2002 12450821 PMC134423

[B10] Bent E Yin B Figueroa A Ye J Fu Q Liu Z Mcdonald V Jeske D Jiang T Borneman J Development of a 9600-clone procedure for oligonucleotide fingerprinting of rRNA genes: Utilization to identify soil bacterial rRNA genes that correlate in abundance with the development of avocado root rot Journal of Microbiological Methods 2006 67 171 180 10.1016/j.mimet.2006.03.023 16712989

[B11] Borneman J Chrobak M Della Vedova G Figueroa A Jiang T Probe selection algorithms with applications in the analysis of microbial communities Bioinformatics 2001 17 Suppl 1 S39 48 10.1093/bioinformatics/17.suppl_1.S39 11472991

[B12] Chung W-H Rhee S-K Wan X-F Bae J-W Quan Z-X Park Y-H Design of long oligonucleotide probes for functional gene detection in a microbial community Bioinformatics 2005 21 4092 4100 10.1093/bioinformatics/bti673 16159916

[B13] Militon C Rimour S Missaoui M Biderre C Barra V Hill D Mone A Gagne G Meier H Peyretaillade E Peyret P PhylArray: phylogenetic probe design algorithm for microarray Bioinformatics 2007 23 2550 2557 10.1093/bioinformatics/btm392 17698494

[B14] Dugat-Bony E Missaoui M Peyretaillade E Biderre-Petit C Bouzid O Gouinaud C Hill D Peyret P HiSpOD: probe design for functional DNA microarrays Bioinformatics 2011 27 641 648 10.1093/bioinformatics/btq712 21216777

[B15] Bader KC Grothoff C Meier H Comprehensive and relaxed search for oligonucleotide signatures in hierarchically clustered sequence datasets Bioinformatics 2011 27 1546 1554 10.1093/bioinformatics/btr161 21471017

[B16] Drmanac R Drmanac S cDNA screening by array hybridization Meth Enzymol 1999 303 165 178 10349645 10.1016/s0076-6879(99)03013-x

[B17] Drmanac S Drmanac R Processing of cDNA and genomic kilobase-size clones for massive screening, mapping and sequencing by hybridization BioTechniques 1994 17 328 329 332-336 7980937

[B18] Meier-Ewert S Lange J Gerst H Herwig R Schmitt A Freund J Elge T Mott R Herrmann B Lehrach H Comparative gene expression profiling by oligonucleotide fingerprinting Nucleic Acids Res 1998 26 2216 2223 10.1093/nar/26.9.2216 9547283 PMC147517

[B19] Yin B Valinsky L Gao X Becker JO Borneman J Bacterial rRNA genes associated with soil suppressiveness against the plant-parasitic nematode Heterodera schachtii Appl Environ Microbiol 2003 69 1573 80 10.1128/AEM.69.3.1573-1580.2003 12620845 PMC150066

[B20] Scupham AJ Presley LL Wei B Bent E Griffith N McPherson M Zhu F Oluwadara O Rao N Braun J Borneman J Abundant and diverse fungal microbiota in the murine intestine Appl Environ Microbiol 2006 72 793 801 10.1128/AEM.72.1.793-801.2006 16391120 PMC1352209

[B21] Ye J Lee JW Presley LL Bent E Wei B Braun J Schiller NL Straus DS Borneman J Bacteria and bacterial rRNA genes associated with the development of colitis in IL-10 Mice Inflamm Bowel Dis 2008 14 1041 1050 10.1002/ibd.20442 18381614 PMC3804113

[B22] Bent E Loffredo A McKenry MV Becker JO Borneman J Detection and Investigation of Soil Biological Activity against Meloidogyne incognita J Nematol 2008 40 109 118 19259527 PMC2586535

[B23] Schloss PD Westcott SL Ryabin T Hall JR Hartmann M Hollister EB Lesniewski RA Oakley BB Parks DH Robinson CJ Sahl JW Stres B Thallinger GG Van Horn DJ Weber CF Introducing mothur: Open-Source, Platform-Independent, Community-Supported Software for Describing and Comparing Microbial Communities Applied and Environmental Microbiology 2009 75 7537 7541 10.1128/AEM.01541-09 19801464 PMC2786419

[B24] Maidak BL Cole JR Parker CT Garrity GM Larsen N Li B Lilburn TG McCaughey MJ Olsen GJ Overbeek R Pramanik S Schmidt TM Tiedje JM Woese CR A new version of the RDP (Ribosomal Database Project) Nucleic Acids Res 1999 27 171 173 10.1093/nar/27.1.171 9847171 PMC148126

[B25] Wang Q Garrity GM Tiedje JM Cole JR Naive Bayesian classifier for rapid assignment of rRNA sequences into the new bacterial taxonomy Appl Environ Microbiol 2007 73 5261 5267 10.1128/AEM.00062-07 17586664 PMC1950982

[B26] Yu H Jeske DR Ruegger P Borneman J Neutral Zone Classifiers Using a Decision-Theoretic Approach With Application to DNA Array Analyses J Agric Biol Environ Stat 2010 15 474 490 10.1007/s13253-010-0034-6 21769245 PMC3137885

