# Supplementary material for: Improving probe set selection for microbial community analysis by leveraging taxonomic information of training sequences
Source: BMC Bioinformatics. 2011 Oct 10;12:394. doi: 10.1186/1471-2105-12-394 (PMC3224148; doi:10.1186/1471-2105-12-394)
Supplement: Additional file 1 — Effect of Sequencing Read Length on Taxonomic Classification Detail. This file contains the detailed results of the simulated read length on taxonomic classification study shown in Figure 8. Simulated reads, of lengths 200 bp up to 1400 bp (in 200 bp increments), were extracted from (already classified) full-length RDP 16S rRNA gene sequences, beginning from several universal bacterial primer sites. Sequences used met the same quality requirements of our data processing pipeline (i.e., they must be of sufficient length and not contain ambiguous bases). For each read length and primer start point, 40, 000 reads were selected randomly and processed through the RDP Classifier (RDPC) version 2.3. We considered a read correctly classified if its classification matched the classification of the full-length sequence from which it came, regardless of the confidence level calculated by the RDPC. [file 1471-2105-12-394-S1.PDF]

	**Read Length**						
**Primer**	200	400	600	800	1000	1200	1400
27F	91.7%	94.5%	96.7%	97.9%	98.6%	98.8%	98.8%
519R	89.8%	94.8%					
530F	90.7%	95.7%	97.1%	98.0%			
907R	88.8%	94.9%	97.2%	98.1%			
926F	87.6%	93.2%					
1100R	86.9%	95.1%	96.9%	97.9%	98.6%		
1114F	87.4%	89.7%					
1492R	84.7%	92.7%	96.0%	97.6%	98.4%	98.7%	98.9%
**Average**	88.4%	93.8%	96.8%	97.9%	98.5%	98.7%	98.9%
